# Adaptation by Ancient Horizontal Acquisition of Butyrate Metabolism Genes in Aggregatibacter actinomycetemcomitans

**DOI:** 10.1128/mBio.03581-20

**Published:** 2021-03-23

**Authors:** Ahmed M. Moustafa, Senthil Kumar Velusamy, Lidiya Denu, Apurva Narechania, Daniel H. Fine, Paul J. Planet

**Affiliations:** a Division of Pediatric Infectious Diseases, Children’s Hospital of Philadelphia, Philadelphia, Pennsylvania, USA; b Department of Oral Biology, Rutgers School of Dental Medicine, Newark, New Jersey, USA; c Sackler Institute for Comparative Genomics, American Museum of Natural History, New York, New York, USA; d Department of Pediatrics, Perelman College of Medicine, University of Pennsylvania, Philadelphia, Pennsylvania, USA; Georgia Institute of Technology School of Biological Sciences

**Keywords:** *Aggregatibacter*, horizontal gene transfer, nutritional immunity, Old World monkey, periodontitis, short-chain fatty acid

## Abstract

Like the bacterial residents of the human gut, it is likely that many of the species in the human oral microbiota have evolved to better occupy and persist in their niche. Aggregatibacter actinomycetemcomitans (*Aa*) is both a common colonizer of the oral cavity and has been implicated in the pathogenesis of periodontal disease. Here, we present a whole-genome phylogenetic analysis of *Aa* isolates from humans and nonhuman primates that revealed an ancient origin for this species and a long history of association with the *Catarrhini*, the lineage that includes Old World monkeys (OWM) and humans. Further genomic analysis showed a strong association with the presence of a short-chain fatty acid (SCFA) catabolism locus (*atoRDAEB*) in many human isolates that was absent in almost all nonhuman OWM isolates. We show that this locus was likely acquired through horizontal gene transfer. When grown under conditions that are similar to those at the subgingival site of periodontitis (anaerobic, SCFA replete), *Aa* strains with *atoRDAEB* formed robust biofilms and showed upregulation of genes involved in virulence, colonization, and immune evasion. Both an isogenic deletion mutant and nonhuman primate isolates lacking the *ato* locus failed to grow in a robust biofilm under these conditions, but grew well under the carbohydrate-rich conditions similar to those found above the gumline. We propose that the acquisition of the *ato* locus was a key evolutionary step allowing *Aa* to utilize SCFAs, adapt, and modulate subgingival disease.

## Abstract (precis)

There has been considerable interest in the impact of short-chain fatty acids (SCFAs) on inflammatory effects related to the microbiome. Here, we present evidence that SCFAs may also be important in disease by providing an energy source or disease-associated cue for colonizing pathogens.

## OBSERVATION

The resident microbiota of mammalian species appears to diversify or “cospeciate” in parallel with the divergence of its hosts (1–5), which results in long-term associations of certain microbial lineages with specific host species. While the biological consequences and driving forces underlying these cophylogenetic patterns remain unclear (3), it is likely that such long-term relationships lead to specific bacterial adaptations to each host species. There is significant evidence supporting bacterial adaptation to the host in the gut (6), but there is little known about the microbiota at other body sites. It is also unclear how adaptation may contribute to health and disease.

Aggregatibacter actinomycetemcomitans (*Aa*) is a Gram-negative oral bacterium that colonizes tooth surfaces in both humans (7) and nonhuman primates (8). However, in humans *Aa* is also strongly associated with localized aggressive periodontitis (LAP) (9), and it is also a known pathogen in endocarditis, brain abscesses, and pneumonia (10). Despite widespread colonization in nonhuman primates, there is no evidence that *Aa* is associated with disease in these animals (11, 12), and highly leukotoxic strains that are strongly associated with disease in humans (13) have not been recovered from primates to date. Together, these observations suggest either that humans are more susceptible to *Aa* virulence or that *Aa* may have evolved to occupy distinct niches in humans and cause disease (14), scenarios that are not mutually exclusive.

*Aa* is thought to be transmitted horizontally by maternal transmission to infants (15–17) and has been found at high rates in young children (e.g., 63% of 6-month-olds [18] and 95.5% of 12-month-olds [19]), with lower rates of colonization in school children (e.g., 13.7% of 11- to 17-year-olds [9]). Moreover, 36% of healthy adults and 50% of those with periodontitis have been reported to carry *Aa* (20). The estimated prevalences of *Aa* in periodontitis are proposed to be 48.1% in patients younger than 35 years and 24.6% in patients older than 35 years (21).

In nonhuman primates, such as rhesus macaques, chimpanzees, bonobos, gorillas, and orangutans, both in captivity and sanctuaries, rates of *Aa* oral colonization vary between 50 and 100% (8, 22), showing that there is a strong affinity for the *Catarrhini* (the group that includes Old World monkeys [OWM], great apes, gibbons, and humans) and contrasting with surveys of New World monkeys, the *Platyrrhini* (8). In addition, several *Aa* adhesins (23, 24) and leukotoxin (LtxA) (25, 26) appear to have specificity for cells from members of the *Catarrhini.* It is notable too that while LtxA is specific for members of the *Catarrhini*, human neutrophils appear to be particularly susceptible to LtxA, even compared to those from close great ape relatives (26).

To understand the diversity of *Aa* strains and evaluate the phylogenetic relationship between isolates from human and nonhuman primates, we produced draft whole-genome sequences (WGSs) for 14 *Aa* isolates (9 from rhesus [Rh] macaques, 2 from green monkeys, 2 from humans, and 1 from a marmoset [see Table S1 in the supplemental material]) and combined these with 81 available *Aa* WGSs from GenBank for comparative analysis.

10.1128/mBio.03581-20.5 TABLE S1 Summary of A. actinomycetemcomitans strains sequenced in this study. Download Table S1, DOCX file, 0.01 MB. Copyright © 2021 Moustafa et al. 2021 Moustafa et al. https://creativecommons.org/licenses/by/4.0/ This content is distributed under the terms of the Creative Commons Attribution 4.0 International license.

**FIG 1:**
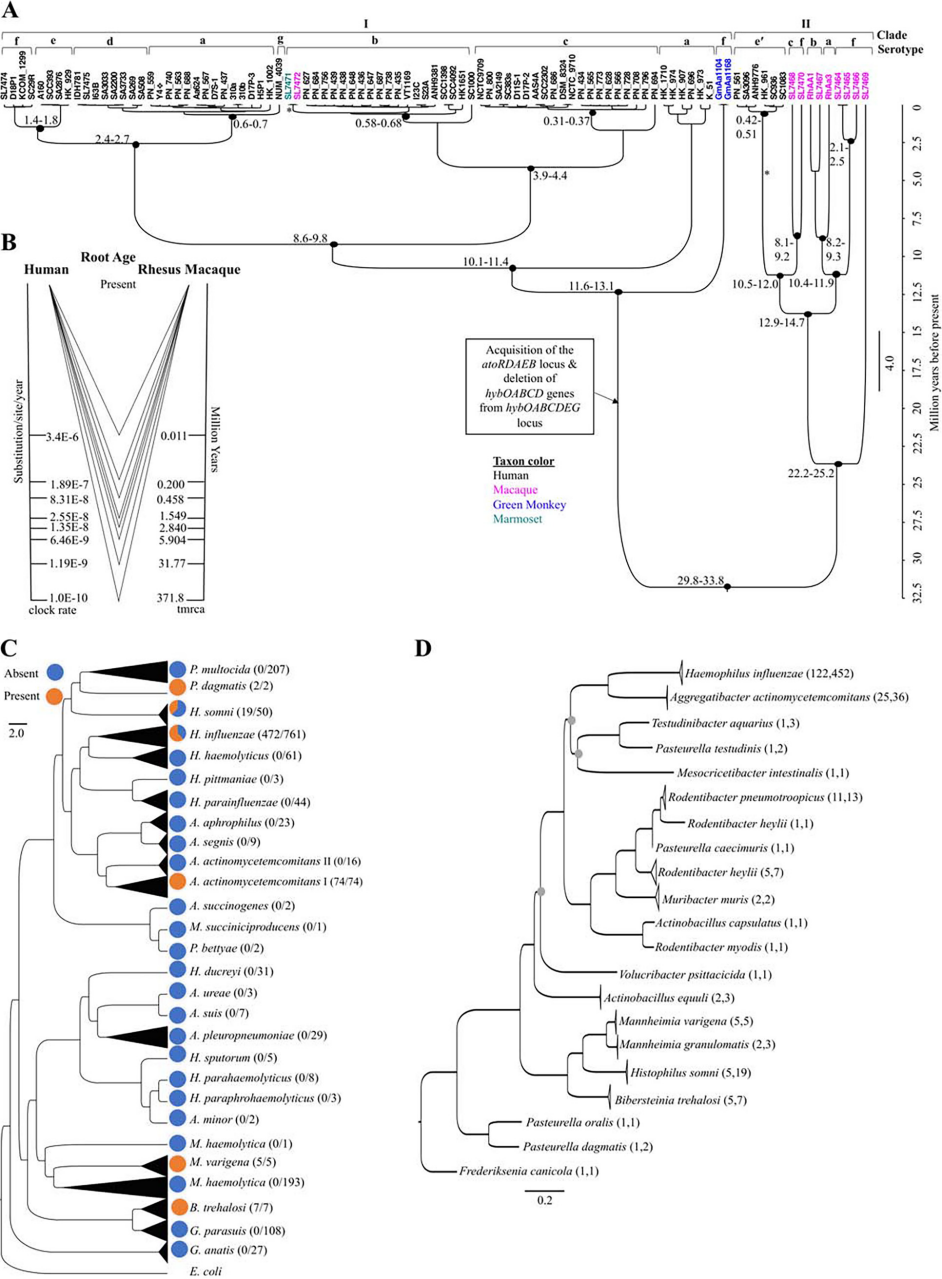
Evolution of A. actinomycetemcomitans and the *atoRDAEB* locus in the *Pasteurellaceae*. (A) A chronogram was constructed using the genome single-nucleotide polymorphisms from 95 *Aa* genomes in a Bayesian phylogenetic analysis that coestimated the phylogenetic relationships among isolates and the time since the divergence. Pictured is an analysis using a substitution rate of 1.19 × 10^−9^ substitutions/site/year that estimates a common ancestor at 31.77 million years ago (mya), with a 95% highest posterior density (HPD) of 29.8 to 33.8 mya. Taxa from humans, macaques, green monkeys, and a marmoset are in black, magenta, blue, and teal, respectively. The 95% HPD is shown beside important nodes. The asterisks on two branches show the interhost transmission (IT) between clades I and II. The black diamond on isolate Y4 represents the discrepancy between the reported serotype in literature and the predicted serotype from the published genome on NCBI. The brackets on branches represent the serotypes a, b, c, d, e, e′, f, and g. Posterior values on all major branches are 1, except for some internal branches. (B) A divergence time triangle diagram shows the different possible divergence times between the two major clades of *Aa* (clade I, composed mainly of human isolates, and clade II, composed mainly of rhesus macaque isolates) on the right axis, with the corresponding evolutionary rates of *Aa* on the left axis. An *Aa* evolutionary rate of 1.19 × 10^−9^ is equivalent to a divergence time close to the MRCA of the *Catarrhini* clade, 32 mya. (C) Phylogenetic analysis of the *Pasteurellaceae* family with pie charts showing the presence or absence of the *atoRDAEB* locus in orange and blue, respectively. The tree was constructed using OrthologID. The total number of genomes for the species present in GenBank that has the locus over the total number of genomes is shown in parentheses. Glaesserella parasuis was previously called Haemophilus parasuis. (D) Phylogenetic analysis of the *atoRDAEB* locus in the *Pasteurellaceae* family. A maximum likelihood tree of the *atoRDAEB* locus in the *Pasteurellaceae* family was created using RAxML. The root was determined using a similar tree that included a close relative (Erwinia teleogrylli) from the *Gammaproteobacteria* as an outgroup. Bootstrap values are 95 to 100 on all branches, except for 3 nodes with gray circles, which have values between 58 and 76. Species clades with more than two genomes were collapsed for easier visualization. The sequences were clustered using CD-HIT (100% identity and coverage). All clusters were composed of individual species. The number of clusters and total number of genomes are shown, respectively, in parentheses.

Whole-genome phylogenetic analysis of the combined data set revealed two major *Aa* clades. Clade I (Fig. 1A) is primarily derived from humans and is divided into subclades that roughly correlate with known serotypes, corroborating prior phylogenetic analyses based on 397 and 1,146 concatenated core genes (27, 28).

Interestingly, clade II expands what is known about a previously described, highly divergent *Aa* lineage that includes human-derived, serotype e′ isolates from Finland and a serotype b *Aa* isolate from an Rh macaque that was previously sequenced by our group (27–30). All but one of the Rh macaque isolates sequenced for this study segregated in this divergent clade II, revealing a strong association of this clade with nonhuman primates.

The added diversity and potentially deep divergence of these strains raised the possibility of a codivergence within the *Aa* species from the most recent common ancestor (MRCA) of the *Catarrhini* approximately 32 million years ago (mya) (31). To test this possibility, we used a whole-genome sequencing (WGS), Bayesian, molecular clock-based approach. There is little information available about the rates of sequence evolution for *Aa*, and in general, estimated rates of bacterial evolution vary greatly (see Text S1 in the supplemental material). Consequently, we decided to take an agnostic, hypothesis-testing approach to infer rates at different putative historical time points for the MRCA of *Aa*. Figure 1B shows a range of rates and the estimated age of the common *Aa* ancestor for each one. With faster evolutionary rates (3.4 × 10^−6^ substitutions/site/year), the divergence between clades I and II was at least 11,000 years old. A reasonably slower evolutionary rate of 1.19 × 10^−9^ showed the divergence time to be close to the MRCA of the *Catarrhini* clade, 32 mya (31), a time point that may signal an *Aa-Catarrhini* “cospeciation” event. However, more sampling from the *Catarrhini* clade would be needed to further support this hypothesis.

10.1128/mBio.03581-20.1 TEXT S1 Detailed methods and extended discussion. Download Text S1, DOCX file, 0.08 MB. Copyright © 2021 Moustafa et al. 2021 Moustafa et al. https://creativecommons.org/licenses/by/4.0/ This content is distributed under the terms of the Creative Commons Attribution 4.0 International license.

To determine differences in genomic content between human and nonhuman strains, predicted protein-coding open reading frames (ORFs) of the assembled genomes were compared among strains using a pangenome approach. There were no genes that were uniquely found in isolates from any particular host species, reflecting the presence of some human and nonhuman isolates across the tree. However, there were a small number of unique and ubiquitous genes defining the two major clades (see Fig. S1 in the supplemental material). Interestingly, all 5 unique genes in clade I mapped to the same locus, the *ato* locus (32) (*atoRDAEB*), which is predicted to be involved in short-chain fatty acid (SCFA) catabolism. The absence of this locus in clade II suggested the possibility that it was acquired by horizontal gene transfer (HGT) in the MRCA of clade I, an event that took place at least 12.4 mya if we assume an *Aa-Catarrhini* codivergence (Fig. 1A). To test this hypothesis, we surveyed all publicly available genomes for this locus from the taxonomic family of *Aa*, the *Pasteurellaceae*, and mapped its presence on a WGS maximum likelihood tree (Fig. 1C). Interestingly, the *ato* locus was absent in all other *Aggregatibacter* species and even closely related *Haemophilus* species, supporting HGT into *Aa* clade I. Notably, the locus was present in 62% of Haemophilus influenzae (*Hflu*) genomes, and 100% of encapsulated *Hflu* strains (see Fig. S2 in the supplemental material), raising the possibility of HGT between *Hflu* and *Aa*. However, a maximum likelihood tree of the *ato* locus sequences themselves (Fig. 1D) showed two distinct *Aa* and *Hflu* clades without one clade nested in the other, which argues against direct HGT between these two species. Given the close relationship between the *Hflu* and *Aa* versions of the locus, it is possible that both species acquired the locus from a common unidentified third organism. Because all of the closest BLAST hits to the *Aa ato* locus were from the *Pasteurellaceae*, and given that the closest relatives from Yersinia intermedia and Morganella morganii fall outside the *Pasteurellaceae* loci, we contend that the most likely donor organism is an unsampled or extinct *Pasteurellaceae* family member (see Fig. S3 in the supplemental material).

10.1128/mBio.03581-20.2 FIG S1 Pangenome analysis of 95 *Aa* isolates from primates. (A) A pie chart of the pangenome, breaking down the genes into core, soft core, shell, and cloud and showing the number of isolates they are present in. (B) A table showing the genes that are unique to the two major clades (Human and Monkey) in the tree of Fig. 1A. (C) Illustration of the genes of the two loci showing the size of each gene in base pairs (bp) and the size of proteins in kDa. Download FIG S1, DOCX file, 0.3 MB. Copyright © 2021 Moustafa et al. 2021 Moustafa et al. https://creativecommons.org/licenses/by/4.0/ This content is distributed under the terms of the Creative Commons Attribution 4.0 International license.

10.1128/mBio.03581-20.3 FIG S2 Phylogenetic analysis of the H. influenzae species showing the presence/absence of the *atoRDAEB* locus and capsule. A maximum likelihood phylogenetic tree was constructed with 755 genomes from GenBank. The first ring and second ring from inside to outside represent the *atoRDAEB* locus and capsule, respectively. All capsulated genomes have the *atoRDAEB* locus. The tree was edited using iTOL website (v4.2.3). The tree scale indicates the number of substitutions per site. Download FIG S2, DOCX file, 2.9 MB. Copyright © 2021 Moustafa et al. 2021 Moustafa et al. https://creativecommons.org/licenses/by/4.0/ This content is distributed under the terms of the Creative Commons Attribution 4.0 International license.

10.1128/mBio.03581-20.4 FIG S3 Phylogenetic analysis of the *atoDAEB* locus in the *Pasteurellaceae* family with outgroup species from the *Gammaproteobacteria*. A maximum likelihood tree of the *atoDAEB* locus in the *Pasteurellaceae* family was created using RAxML with *Gammaproteobacteria* species *Y. intermedia*, M. morganii, and Erwinia teleogrylli included as outgroups. The root was determined on the branch that makes the *Pasteurellaceae* family an ingroup and other members from the *Gammaproteobacteria* an outgroup. Bootstrap values are reported on all branches. Species clades with more than four clusters were collapsed for easier visualization. The alignment was limited to *atoDAEB*, *atoR* was excluded as it is absent outside the *Pasteurellaceae* family. The sequences were clustered using CD-HIT (100% identity and coverage). All clusters were composed of individual species. The total number of genomes in each cluster and sequence length are shown, respectively, and separated by underscore in the nodes that were not collapsed. Download FIG S3, DOCX file, 0.02 MB. Copyright © 2021 Moustafa et al. 2021 Moustafa et al. https://creativecommons.org/licenses/by/4.0/ This content is distributed under the terms of the Creative Commons Attribution 4.0 International license.

**FIG 2:**
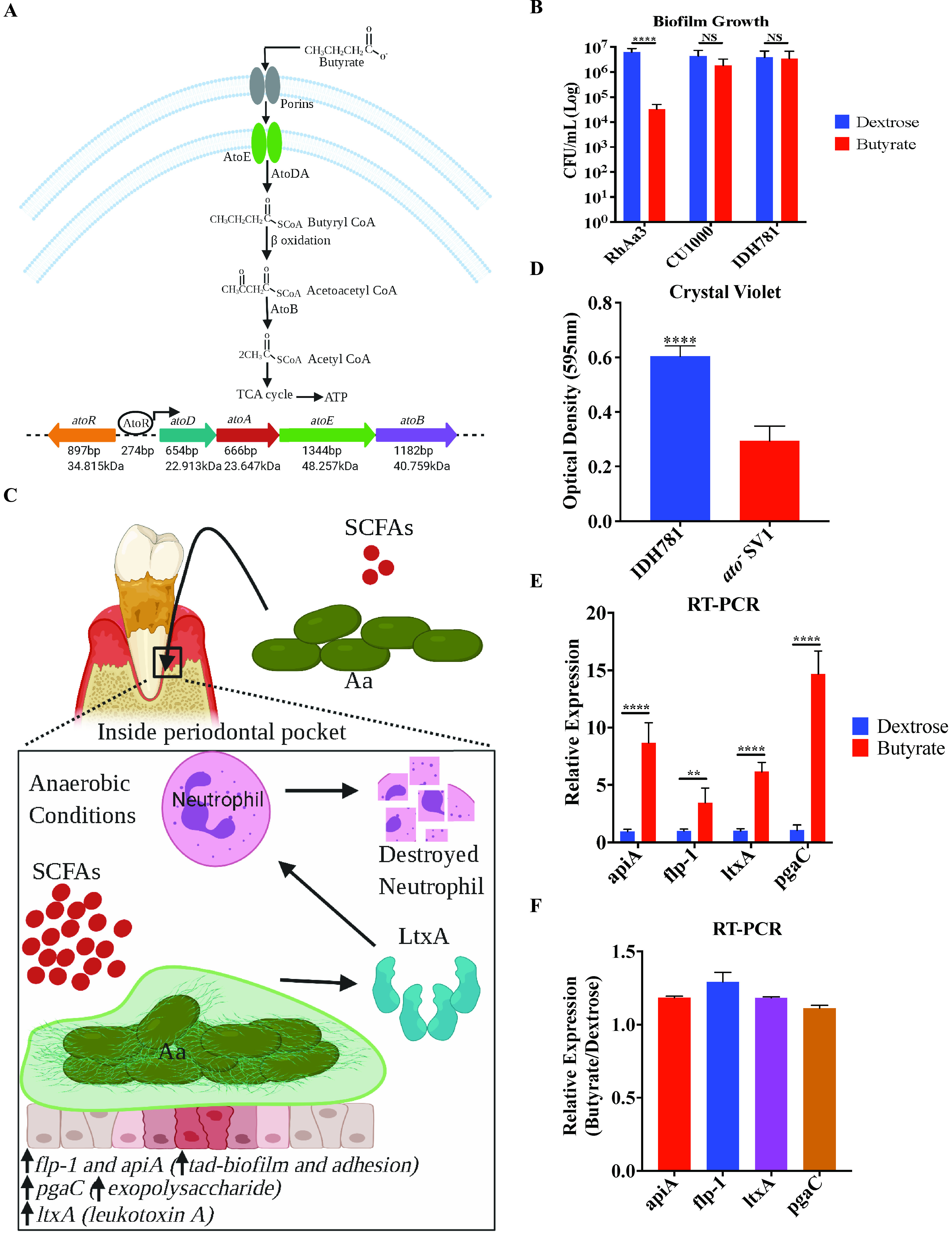
Requirement of the *ato* locus for utilization of SCFAs and increased transcription of virulence genes. (A) Model for catabolism of butyrate by the *atoRDAEB* locus in A. actinomycetemcomitans (*Aa*). The AtoR protein induces *atoDAEB* locus transcription by binding to the promoter. Butyrate is transported through the cytoplasmic membrane by the AtoE membrane transporter and is then converted to acetyl coenzyme A (acetyl-CoA) by the *atoDA*-encoded acetyl-CoA:acetoacetyl-CoA transferase and the *atoB*-encoded acetoacetyl-CoA thiolase. (B) Biofilm growth of two human strains (IDH781 and CU1000) from clade I and a rhesus macaque strain (RhAa3) from clade II on two different carbon sources, dextrose and butyrate. The biofilm growth in CFU is shown on the *y* axis. Although the Rh macaque strain has a significantly reduced CFU count from biofilm on butyrate compared to dextrose, it should be noted that there is only negligible planktonic growth in either condition. Thus, the large majority of growth is in the biofilm. Significance was calculated with a *t* test. (C) Working model for *Aa* outside and inside the periodontal pocket. Once inside the periodontal pocket, *Aa* utilizes SCFAs (e.g., butyrate) as a carbon source, and it increases *tad* biofilm, adherence, and exopolysaccharide and toxin production. (D) Crystal violet biofilm assay of the human strain IDH781 and a derived *ato* mutant strain on butyrate as a carbon source. The optical density of destaining fluid (595 nm) is shown on the *y* axis. Significance was calculated with a *t* test. (E) RT-PCR of different *Aa* virulence genes in the human strain IDH781. Relative expression is presented normalized to mean 16S rRNA ± standard error of the mean (SEM) for 6 separate cultures. (F) RT-PCR of the same relative expression data shown in panel E, but this time normalizing to the average transcript level for each gene in dextrose. The total starting RNA from dextrose and butyrate biofilm cells were equal before cDNA synthesis. Results are presented as the mean ± SEM from 6 separate cultures. The genes *apiA*, *flp-1*, *ltxA*, and *pgaC* code for adhesion, *tad* biofilm, toxin, and exopolysaccharides, respectively. A *P* value of ≤0.05 was considered significant. The asterisks represent *P* values of ≤0.01 (**) and ≤0.0001 (****). NS, not significant.

Based on work in Escherichia coli (32–35), we hypothesized that *Aa* with the *ato* locus would be able to catabolize the SCFA butyrate and use it as a carbon source or as a cue to enhance colonization (Fig. 2A). In media with butyrate as a defined carbon source, strains containing the *ato* locus grew significantly better and formed more robust biofilms than macaque-derived *Aa* strains lacking the *ato* locus as well as an isogenic human-derived Δ*ato* strain (Fig. 2B and D). Differences in pH between the different media tested were minimal (see Text S1, Table S2, and Table S3 in the supplemental material).

10.1128/mBio.03581-20.6 TABLE S2 Medium pH after adding dextrose and butyrate as carbon sources. Download Table S2, DOCX file, 0.01 MB. Copyright © 2021 Moustafa et al. 2021 Moustafa et al. https://creativecommons.org/licenses/by/4.0/ This content is distributed under the terms of the Creative Commons Attribution 4.0 International license.

10.1128/mBio.03581-20.7 TABLE S3 Primers used in the deletion of the *ato* locus. Download Table S3, DOCX file, 0.01 MB. Copyright © 2021 Moustafa et al. 2021 Moustafa et al. https://creativecommons.org/licenses/by/4.0/ This content is distributed under the terms of the Creative Commons Attribution 4.0 International license.

Interestingly, the only other whole-gene event associated with clade I is a deletion of several genes in the *hybOABCD* locus (Fig. S1). The products of this locus are known to metabolize H_2_ under anaerobic conditions, using it as an electron donor for respiration and thus energy production (36, 37). We speculate that the production of protons by the Hyb system might exacerbate the toxic properties of butyrate or that acquisition of the Ato system made the energy-generating properties of the Hyb system redundant (Text S1). However, the interplay between the acquisition of the *ato* locus and the deletion of the *hyb* locus remains to be explored in future studies.

We next hypothesized that *ato*-harboring isolates may be uniquely adapted to utilize SCFAs in the human subgingival periodontal niche, since microbes that produce SCFAs are elevated at this site during *Aa* infection (38). The subgingival niche is highly anaerobic and has few energy sources other than amino acids and butyrate (39, 40).

Because the anaerobic subgingival pocket is the site of periodontitis, we hypothesized that *Aa* would respond to high levels of butyrate by upregulating genes associated with virulence. Using reverse transcription-quantitative PCR (RT-qPCR), we showed that *Aa* grown in butyrate, under anaerobic conditions, transcriptionally upregulated a leukotoxin (*ltxA*) gene that can kill activated neutrophils, the epithelial adhesin *apiA* gene, the biofilm/adherence gene *flp-1*, and the exopolysaccharide gene *pgaC* (Fig. 2C, E, and F).

The anti-inflammatory properties of SCFAs in the gut are well documented (41, 42), but the studies considering the periodontal pocket suggest that they may be proinflammatory and may worsen the disease (43–46). Thus, the impact of *Aa* butyrate catabolism on disease modulation is unclear. Interestingly, the four Finnish strains from clade II that do not have the *ato* locus appear to have been obtained from “healthy or minimally diseased individuals”(15–17).

In conclusion, the present study shows a higher level of genomic diversity in *Aa* than previously recognized and highlights a major clade dominated by isolates from rhesus macaques, raising the possibility of a long history of primate-*Aa* codiversification. Based on our molecular clock analysis, we favor the hypothesis that the MRCA of *Aa* was present in the MRCA of the *Catarrhini* about 32 mya. We propose that after this codivergence event, the lineage that would eventually dominate in humans acquired the *ato* locus, allowing it to more successfully colonize the periodontal pocket replete with butyrate and modulate the host response to disease.

### Data availability.

The genome sequences and the reads have been submitted to GenBank and SRA under BioProject PRJNA641505. Accession numbers are available in Table S1. The raw trees and the Roary gene presence report are available to download from reference 47.
