# Supplementary material for: Adaptation by Ancient Horizontal Acquisition of Butyrate Metabolism Genes in Aggregatibacter actinomycetemcomitans
Source: mBio. 2021 Mar 23;12(2):e03581-20. doi: 10.1128/mBio.03581-20 (PMC8092312; doi:10.1128/mBio.03581-20)
Supplement: TABLE S1 [file mBio.03581-20-st001.docx]

**Summary of *A. actinomycetemcomitans* strains sequenced in this study.**

| **Isolate** | **Host** | **Serotype** | **GenBank** | **BioSample** | **SRA** | **Genome size (Mb)** | **GC content** | **Location** |
| --- | --- | --- | --- | --- | --- | --- | --- | --- |
| SL7464 | *Macaca mulatta* | F | JACAWL000000000 | SAMN15355013 | SRR12084743 | 2.34 | 44.2 | NEPRC |
| SL7465 | *Macaca mulatta* | F | JACAWK000000000 | SAMN15355014 | SRR12084742 | 2.34 | 44.2 | NEPRC |
| SL7466 | *Macaca mulatta* | F | JACAWJ000000000 | SAMN15355015 | SRR12084737 | 2.33 | 44.1 | NEPRC |
| SL7467 | *Macaca mulatta* | B | JACAWI000000000 | SAMN15355016 | SRR12084736 | 2.34 | 44.3 | NEPRC |
| SL7468 | *Macaca mulatta* | F | JACAWH000000000 | SAMN15355017 | SRR12084735 | 2.40 | 44.2 | IACUC |
| SL7469 | *Macaca mulatta* | F | JACAWG000000000 | SAMN15355018 | SRR12084734 | 2.61 | 45.9 | IACUC |
| SL7470 | *Macaca mulatta* | C | JACAWF000000000 | SAMN15355019 | SRR12084733 | 2.33 | 44.1 | IACUC |
| SL7471 | *Callithrix jacchus* | B | JACAWE000000000 | SAMN15355020 | SRR12084732 | 2.24 | 44.2 | SNPRC |
| SL7472 | *Macaca mulatta* | B | JACAWD000000000 | SAMN15355021 | SRR12084731 | 2.24 | 44.2 | SNPRC |
| SL7474 (CU1000) | *Homo sapiens* | F | JACAWC000000000 | SAMN15355022 | SRR12084730 | 2.26 | 44.1 | Columbia University, NY |
| SL7475 (IDH781) | *Homo sapiens* | D | JACAWB000000000 | SAMN15355023 | SRR12084741 | 2.22 | 44.2 | Columbia University, NY |
| RhAa3 | *Macaca mulatta* | A | JACAWA000000000 | SAMN15355024 | SRR12084740 | 2.50 | 44.3 | NEPRC |
| Gm1104 | *Chlorocebus sabaeus* | F | JACAVZ000000000 | SAMN15355025 | SRR12084739 | 2.52 | 44.1 | SNPRC |
| Gm1168 | *Chlorocebus sabaeus* | F | JACAVY000000000 | SAMN15355026 | SRR12084738 | 2.51 | 44.1 | SNPRC |
