# Supplementary material for: Adaptation by Ancient Horizontal Acquisition of Butyrate Metabolism Genes in Aggregatibacter actinomycetemcomitans
Source: mBio. 2021 Mar 23;12(2):e03581-20. doi: 10.1128/mBio.03581-20 (PMC8092312; doi:10.1128/mBio.03581-20)
Supplement: TEXT S1 [file mBio.03581-20-s0001.docx]

Table of Contents

[Detailed Methods 2](#_Toc59142678)

[1. Bacterial strains 2](#_Toc59142679)

[2. Genomic Sequencing and Assembly. 2](#_Toc59142680)

[3. Establishment of Gene Orthology and Presence/Absence Heatmap Matrix 2](#_Toc59142681)

[4. Divergence time analysis 2](#_Toc59142682)

[5. Pasteurellaceae and ato locus trees 3](#_Toc59142683)

[6. Biofilm growth of A. actinomycetemcomitans strains 3](#_Toc59142684)

[7. Detailed instructions for broth medium for A. actinomycetemcomitans 3](#_Toc59142685)

[8. RNA extraction and qRT-PCR 7](#_Toc59142686)

[9. Construction of ato deletion strain 8](#_Toc59142687)

[Extended Discussion 9](#_Toc59142688)

[1. Divergence time analysis 9](#_Toc59142689)

[2. Aa colonization 9](#_Toc59142690)

[3. The acquisition of the ato locus and the deletion of the hyb locus 9](#_Toc59142691)

[References 11](#_Toc59142692)

# Detailed Methods

# Bacterial strains

The Institutional Animal Care and Use Committees (IACUC) at Rutgers University, the New England Primate Research Center (NEPRC) at Harvard Medical School and the Southwest National Primate Research Center (SNPRC) approved all work with primates. IDH781, CU1000 & RhAa3 are from the lab stock of oral biology, RSDM, Newark. Fourteen *Aa* isolates, 9 Rhesus monkey isolates, 2 green monkey isolates, 1 marmoset isolate and 2 human isolates were obtained (Supplementary Table 1). The twelve non-human primate *Aa* strains were isolated from plaque obtained from each of the primates using a cyto-brush collection method. The bacteria in the cyto-brush was suspended in 1 ml of PBS and serially diluted samples were plated on to AAGM supplemented with bacitracin and vancomycin. Plates were incubated for 24-48 h in a 37°C incubator under 10% CO2 atmosphere. *Aa* on the plates was identified based on its specific characteristic star shaped colonial morphology. Selected colonies were tested for catalase activity and final confirmation was carried out by PCR using *ltxA* specific primer(1).

# Genomic Sequencing and Assembly.

Genomic DNA of the eleven isolates was isolated using DNAesy kit (Qiagen). Paired-end 101-bp sequencing protocol on an Illumina HiSeq 2500 was used. The genomes were assembled by ABySS v1.3.5(2). The genome sequences and the reads were submitted to GenBank and SRA under BioProject PRJNA641505. Accession numbers are available in Supplementary Table 1.

# Establishment of Gene Orthology and Presence/Absence Heatmap Matrix

Orthologous gene sets were determined from 95 assembled isolates’ genomes, using both modified version of the OrthologID pipeline(3), that now uses OrthoMCL(4) for gene family clustering, and Roary (v3.12.0)(5). For the pangenome analysis, genomes were first annotated using RAST(6-8) and Prokka (v1.13)(9) for OrthoMCL and Roary, respectively. The presence and absence of genes was determined from the resulting concatenated alignment matrix of orthologs, and then were compared to unique genes predicted by Roary. For simplicity, and to focus on proteins of known functions, hypothetical proteins less than 50 amino acids were excluded from the unique genes list. A multiFASTA alignment of core genes was created using the -e option in Roary, PRANK alignment (10).

# Divergence time analysis

Phylogenetic analysis was done using the multiFASTA alignment created with Roary in a Bayesian framework with BEAST 2.4.7 (11). The JC69 nucleotide substitution model(12) was used. A constant-size coalescent tree process was assumed. A strict molecular clock was used. Each run of the Markov chain Monte Carlo (MCMC) procedure consisted of 12 million steps with a 1000-step thinning. After inspection of the MCMC traces and the effective sample size (ESS) values of each run (ESS > 200), 10% of the first posterior samples were removed as a burn-in. A range of possible evolutionary rates was considered for the analysis. The chronogram was plotted on the basis of the maximum clade credibility tree using the TreeAnnotator program from the BEAST package. The program FigTree v1.4.3 (available at: http://tree.bio.ed.ac.uk/ software/figtree/) was used to make Figure 1A.

# Pasteurellaceae and *ato* locus trees

A modified version of the OrthologID pipeline(3) was used to identify the pangenome and construct an alignment. A maximum likelihood phylogenetic tree for the *Pasteurellaceae* family was then constructed using RAxML v8.2.4 (13). Pie charts representing presence and absence of the *atoRDAEB* locus in the different species of the *Pasteurellaceae* family were constructed using blastn (identity and coverage > 90%). The *atoRDAEB* locus sequence present in all members of the *Pasteurellaceae* family were aligned using MAFFT(14) and clustered using CD-HIT(15) (100% identity and coverage). A maximum likelihood phylogenetic tree was then constructed using RAxML v8.2.4 (13).

# Biofilm growth of *A. actinomycetemcomitans* strains

*A. actinomycetemcomitans* strains were streaked on BHI plates and allowed to grow for 24 hours in a 37 °C incubator at 10% CO_2_ atmosphere. Colonies were scrapped and suspended in BHI broth. The auto-aggregated cells were disrupted using a handheld homogenizer (Kimble Chase, Vineland, NJ) and the non-aggregated free cells were removed by leaving the suspension on ice for two minutes. The cell density of the top portion was adjusted to ~10^8^ cells per mL (OD600 = 0.7–0.8). These cells were used as the inoculum for biofilm growth. Biofilms were grown in broth media supplemented with 40mM dextrose and or butyrate along with 5% of peptone (16) to promote growth for 48 hr on 100 mm by 15 mm Petri dish plates in an anaerobic incubator containing 80% N_2_, 10% CO_2_ and 10% H_2_. After 48 hours of growth in the anaerobic chamber, cells attached to the bottom of the plates were harvested by scraping from the plate and completely suspended in 1 mL of BHI broth. These cells were serially diluted and plated on to BHI agar plates. The colonies enumerated after 48 hours represent biofilm growth on each plate as derived from broth containing butyrate or dextrose. Data are derived from triplicate experiments and expressed as CFU/mL in butyrate and/or dextrose, plotted on the y-axis. Significance was calculated by one-way ANOVA with Tukey’s post-hoc multiple comparison test in GraphPad Prism v7. A *P<0.05 was considered significant.

# Crystal Violet Assay

The crystal violet staining was performed as described previously (17). The biofilm cells attached on the 96 well plates were washed in distilled water for 3 times and the plates were air dried in a 37 °C incubator. The wells were loaded with 200ul of Gram Crystal violet (Thermo Fisher R40052) and incubated in room temperature for 1 hr. Then the plates were washed three times and air dried. The bound crystal violet was suspended in 35% glacial acetic acid and absorbance of the suspension solution was read at OD545 nm. Data presented were the biological triplicates from at least 9 wells.

# Detailed instructions for broth medium for *A. actinomycetemcomitans*

1. Make 20X amino acid/purine/pyrimidine stock in 100 ml of H_2_O with NaOH, follow chart:

| **Amino Acids** | 1L of 20X (g) | 100ml of 20X, mg |
| --- | --- | --- |
| L-Glutamic acid HCl * | 4.9920 | 499.2 |
| DL-Alanine | 2.0000 | 200 |
| L-Leucine | 2.0000 | 200 |
| Glycine | 2.0000 | 200 |
| L-Valine | 2.0000 | 200 |
| L-Tryptophan | 2.0000 | 200 |
| L-Threonine | 2.0000 | 200 |
| L-Serine | 2.0000 | 200 |
| L-Lysine HCl | 2.0000 | 200 |
| L-Arginine HCl * | 2.4200 | 242 |
| L-Histidine HCl H2O* | 2.7020 | 270.2 |
| L-Glutamine | 2.0000 | 200 |
| L-Asparagine H2O * | 2.2720 | 227.2 |
| L-Methionine | 2.0000 | 200 |
| L-Isoleucine | 2.0000 | 200 |
| L-Proline | 2.0000 | 200 |
| L-Aspartic acid | 2.0000 | 200 |
| L-Phenylalanine | 2.0000 | 200 |
| L-Tyrosine | 0.4000 | 40 |
| L-Cystine | 0.1000 | 10 |
| L-Ornithine HCl | 0.4000 | 40 |
| L-Hydroxyproline | 0.4000 | 40 |

| **Purines/Pyrimidines** | 1L of 20X | 100 ml of 20X, (mg) |
| --- | --- | --- |
| Adenine | 0.2500 | 20 |
| Guanine | 0.2000 | 20 |
| Cytosine HCl* | 0.2700 | 27 |
| Thymine | 0.2000 | 20 |
| Xanthine | 0.2000 | 20 |
| Hypoxanthine | 0.2000 | 20 |
| Uracil | 0.2000 | 20 |

** Store the filter sterilized amino acid stock at 4ºC, wrapped in foil.

1. Make 20X inorganic salt stock in 99.5 ml of water, follow chart:

| **Inorganic salts** | 1L of 20X (g) | 100ml of 20X, mg |
| --- | --- | --- |
| MnSO_4_ | 0.1000 | 10 |
| NaCl | 2.0000 | 200 |
| K_2_HPO_4_ | 4.0000 | 400 |
| KH_2_PO_4_ | 20.0000 | 2000 |
| KNO3 | 2.0000 | 200 |

1. Make 20,000X individual stocks of the following salts, follow chart:

| **Inorganic salts** | 1L of 20X (g) | 100ml of 20X,  (g) | mg/ml | 10 ml of 20,000X, (mg) |
| --- | --- | --- | --- | --- |
| KI | 0.0020 | 0.0002 | 0.002 | 20 |
| CuSO_4._5H_2_O * | 0.0013 | 0.00013 | 0.0013 | 13 |
| Boric acid | 0.0100 | 0.001 | 0.01 | 100 |
| ZnSO_4_.7H_2_O * | 0.0140 | 0.0014 | 0.014 | 140 |
| Sodium molybdate | 0.0100 | 0.001 | 0.01 | 100 |

Add these stocks to the inorganic salt stock from above, (100 ul for 100 ml).

** Store the filter sterilized inorganic salt stock at room temperature.

1. Make a 1000X solution of water soluble vitamin stock in 10 ml of water.

| Vitamins/factors | 1L of 1000x, (g) | 10 ml of 1000X, (mg) |
| --- | --- | --- |
| Choline chloride | 50.0000 | 500 |
| Beta-alanine | 10.0000 | 100 |
| Pyridoxal | 1.0000 | 10 |
| Pyridoxine HCl | 1.0000 | 10 |
| Pyridoxamine diHCl | 1.0000 | 10 |
| Spermidine triHCl | 1.0000 | 10 |
| Nicotinic acid | 1.0000 | 10 |
| Nicotinamide | 1.0000 | 10 |
| Calcium pantothenate | 1.0000 | 10 |
| Spermine tetraHCl | 1.0000 | 10 |
| Thiamine HCl | 1.0000 | 10 |
| myo-Inositol | 10.0000 | 100 |
| Nicotinamide adenine dinucleotide | 1.0000 | 10 |
| *p*-Aminobenzoic acid | 0.1000 | 1 |

1. Make a 1000X solution to add to the 1000X vitamin solution (1,000,000X), follow chart:

| **Vitamins/factors** | 1000X-1L (g) | 1000X -10 ml,  (mg) | 1000X,  mg/ml | 10 ml 1000000X,  (mg) |
| --- | --- | --- | --- | --- |
| Vitamin B_12_ | 0.0100 | 0.1 | 0.01 | 100 |

Add this to the 1000X water soluble vitamin stock (10 μl in 10 ml vitamin solution).

** Store water soluble vitamin stock at 4ºC, wrapped in foil to protect from light.

1. Make a 20X stock solution of NaHCO_3_ in 10 ml of water, follow chart:

** Store NaHCO_3_ at room temperature.

|  | 1L of 20X | 100 ml of 20X, (g) | 10 ml of 20X, (mg) |
| --- | --- | --- | --- |
| NaHCO_3_ | 20.0 | 2 | 200 |

1. Make a 100X stock solution of L-cysteine in 10 ml of water, follow chart:

|  | 1L of 20X | 100 ml of 20X, (g) | 10 ml of 100X, (mg) |
| --- | --- | --- | --- |
| L-Cysteine HCl* | 13.0 | 1.3 | 650 |

8. Make a 100X solution of MgSO_4_ in 10 ml of water, follow chart:

**Store at room temp.

|  | 1L of 1X (g) | 10ml of 1X, (mg) | 10ml of 100X, (mg) |
| --- | --- | --- | --- |
| MgSO_4_.7H_2_O | 0.700 | 7 | 700 |

9. Make a 1000X solution of FeSO_4_ in 10 ml of water, follow chart:

**Store at room temp, make fresh every week or after ppt.

|  | 1L of 1X (mg) | 10ml of 1X, (mg) | 10ml of 1000X, (mg) |
| --- | --- | --- | --- |
| FeSO_4_.7H_2_O | 5 | 0.05 | 50 |

10. Make a 100X solution of CaCl_2_ in 10 ml of water, follow chart:

**Store CaCl_2_ at room temp, and do not add to cold medium or it will ppt!

|  | 1L of 1X (mg) | 10ml of 1X, (mg) | 10ml of 100X, (mg) |
| --- | --- | --- | --- |
| CaCl_2_.2H_2_O | 100 | 1 | 100 |

11. Make a 1000X solution of pimelic acid and D-Biotin in 10 ml 50% ethanol, follow chart:

** Store the pimelic acid/D-Biotin stock solution at 4ºC.

|  | 1L of 1X (mg) | 10ml of 1X, (mg) | 10ml of 1000X, (mg) |
| --- | --- | --- | --- |
| Pimelic acid | 0.1000 | 0.001 | 1 |
| D-Biotin | 0.1000 | 0.001 | 1 |

1. Make a 100X solution of riboflavin in 10 ml of warm water, follow chart:

**Store at 4ºC, wrapped in foil to protect from light.

|  | 1L of 1X (mg) | 10ml of 1X, (mg) | 10ml of 100X, (mg) |
| --- | --- | --- | --- |
| Riboflavin | 1 | .01 | 1 |

13. Make a 1000X solution of DL-6,8-thioctic acid in 10 ml of

10% β-mercaptoethanol, 90% ethanol solution, follow chart:

**Store the DL-6,8-thioctic acid solution at 4ºC.

|  | 1L of 1X (mg) | 10ml of 1X, (mg) | 10ml of 1000X, (mg) |
| --- | --- | --- | --- |
| DL-6,8-thioctic | 0.1 | .01 | 1 |

1. Make a 1000X solution of folic acid in 9.975 ml water with 25 μl of 14.8N NH_4_OH, follow chart:

|  | 1L of 1X (mg) | 10ml of 1X, (mg) | 10ml of 1000X, (mg) |
| --- | --- | --- | --- |
| Folic acid | 1 | .01 | 10 |

Steps for preparing 100 mL of *Aa* Medium:

1. Add these amounts:

| Amount (mL) |  |
| --- | --- |
| 5 | Amino acids/Nucleotides (20X) |
| 5 | Inorganic Salts (20x) |
| 1 | L-Cysteine (100X) |
| 1 | Riboflavin (100x) |
| 0.1 | Pimelate/Biotin (1000x) |
| 0.1 | Folic Acid (1000x) |
| 0.1 | Soluble Vitamins (1000x) |
| 0.1 | Thioctic Acid (1000x) |
| 2 | 1M MOPS (20mM Final concentration) |
| 1 mg/mL | NaHCO3 (Add as powder to final concentration listed) |
| 14.4 | Total Volume |

1. Water 81.5 (Add about 5-10 mL less than this to leave room to pH).
2. pH Medium here to 7.2.
3. 40mM Final concentration of glucose or other carbon source can be added).
4. Filter sterilize.
5. Add appropriate amounts of Mg, Ca, and Fe only when ready to use CDM

| Amount (mL) |  |
| --- | --- |
| 1 | MgSO4 (100x) |
| 1 | CaCl2 (100x) |
| 0.1 | FeSO4 (1000x) |

# RNA extraction and qRT-PCR

Biofilm cells of IDH781 were grown as described above in a medium supplemented with dextrose and butyrate for 36 hr. The cells were washed three times with sterile PBS and scraped from the plates. The auto-aggregated cells were completely suspended in the suspension buffer using a handheld homogenizer (Kimble Chase, Vineland, NJ). Total RNA extraction was carried out as previously described(18). The RNA was purified using Micro Bio-Spin P-30 Gel Columns (Bio-Rad, Hercules, CA) and treated with DNaseI and a RNA purification kit (Zymo Research, Irvine) to remove genomic DNA contamination. PCR was performed with Taq DNA polymerase by using *apiA* primers to confirm the elimination of genomic DNA before proceeding to cDNA conversion. Total RNA (~1μg) was converted into cDNA in the first step using High Capacity cDNA kit as described in the manufacturer’s instruction (Applied Biosystems, Foster City, CA). To test that *Aa* transcriptionally upregulates leukotoxin (*ltxA*) that can kill activated neutrophils(19), the epithelial adhesin *apiA*(20)*,* biofilm/adherence gene *flp-1*(21), and exo-polysaccharide gene *pgaC*(22), a second PCR step was carried out with selected gene primers (*apiA*, *flp-1*, *ltxA* and *pgaC*) using cDNA template. qRT-PCR was carried out with Roche SYBR green master mix in a LightCycler480 system. A 25 μl qPCR reaction using the cDNA template was performed as described in the user manual (Roche Life Science, Indianapolis, IN). 16s rRNA gene was used as the internal normalization control. Melting curve analysis was performed to analyze the specificity of the amplified product. Data analysis was performed using LightCycler 480 software (Version 1.2.9.11). A reaction without reverse transcriptase was always performed as a negative control. Results were shown as the standard errors of the means (±SEM) calculated from independent triplicate experiments. Data was analyzed by Students t test. A P<0.05 was considered significant.

# Construction of *ato* deletion strain

A scarless, marker-less deletion approach was used to construct *ato* isogenic mutant from IDH781 strain as described previously (23). Primer pairs atoNotIUF, atoUR, atoDF, and atoXhoIR (Supplementary Table 3) were used to amplify the upstream and downstream flanking regions of the *ato* locus (Accession: CP016553.1, 7201 bp deletion of ORFs including *atoRDAEB*) to be deleted using IDH781 genomic DNA as the template. Both the upstream and downstream fragments were amplified with 15 bp complementary to each other to enable fusion between the fragments. Besides, partial restriction sites *NotI and XhoI* restriction sites were included in 5′ and 3′ ends of the flanking fragments respectively by PCR to enable infusion cloning into pJT1. The first overlap extension PCR (OEPCR) was performed as described previously (24) with the equimolar concentrations of the upstream and downstream flanking fragments without primers. The second PCR was performed using atoNotIF and atoXhoIR primers with 5 µl template from the first OEPCR. The fused PCR amplified fragment was then ligated into NotI-XhoI double digested pJT1 plasmid using in-fusion cloning strategy (Takara Inc., Shiga, Japan). The resultant *ato* deletion plasmid was designated pSV7. The plasmid was confirmed by restriction digestion and sequencing. The plasmid pSV7 was then electroporated into IDH781 and the spectinomycin resistant transformants were replicated on BHI plates supplemented with sucrose (10% w/v) to screen the double cross over strains. Then, spectinomycin sensitive, sucrose resistant colonies were screened by PCR using primers atoOUTF and atoOUTR as described previously (23). The resultant *ato* mutant strain was designated as SV1. The final *ato* deletion strain SV1 was confirmed by sequencing the PCR product obtained from amplicon of atoOUTF and atoOUTR primers.

# Extended Discussion

# Divergence time analysis

There is little information available about the rates of sequence evolution for *Aa* and, in general, estimated rates of bacterial evolution vary greatly (25, 26). For instance, long-term evolutionary rates over millions of years have been estimated to be between 1x10^-9^ to 1x10^-10^ substitutions per site per year (s/s/y)(26-29) with observed short term rates over months or years are between 1x10^−5^ to 10^−7^ (26, 30-33). Haubeck et al(34) used a mutation rate of 1x10^-10^ s/s/y based on reported *E. coli* rates to estimate the age of the JP2 clone of *Aa* (2,400 years ago)*.* Consequently, we decided to take an agnostic, hypothesis testing approach to infer rates at different putative historical time points for the most recent common ancestor (MRCA) of *Aa*.

Very fast rates that are not usually observed in bacteria (10^-4^-10^-5^ s/s/y) would be required to have an *Aa* MRCA in the last 100-1000 years, suggesting that we can reasonably reject our null hypothesis that primates acquired their colonizing strains from humans in captivity. However, our phylogeny does suggest that there has been transmission of lineages found in humans to non-human primates and vice versa. Two isolates, one from a marmoset and one from a rhesus macaque (SL7471 and SL7472) are found in the mainly-human Clade I, and four human isolates are found in the mainly-non-human Clade II. It is unclear whether these represent transmission events that occurred during primate captivity because of our uncertainty about the age of the MRCA. However, if we assume an *Aa/Catarrhini* co-divergence, the putative human-to-monkey event occurred 0.54 million years ago, and the monkey-to-human 11.22 million years ago, suggesting transmission between primate ancestors or other hosts. Interestingly, the two green monkey isolates add a new deeply diverging lineage, and the earliest known branch of Clade I. These isolates could signal that even greater diversity may be found with further sampling from the *Catarrhini*.

# *Aa* colonization

When *Aa* initially colonizes the supragingival environment it prefers lactate as its chief carbon or energy source(35), which is a byproduct of metabolism of glucose by early colonizers such as oral streptococci. While this preference frees *Aa* from competition with other pioneer colonizers for a carbon source, streptococci also produce H_2_O_2_ that is toxic to *Aa*(36). Under this pressure, *Aa* upregulates dispersinB (DspB), which acts on its biofilm exopolysaccharide (EPS), allowing it to enter the subgingival environment(37). *Aa* has also been shown to interact *in vivo* with other subgingival bacteria that are known to make SCFAs(37).

# The acquisition of the *ato* locus and the deletion of the *hyb* locus

The pH of the subgingival anaerobic environment is acidic due to fermentative acids produced by the bacterial community, and it also has high levels of SCFAs (particularly butyrate). In acidic environments, butyrate can be problematic due to its weak acidic nature (the pKa of butyrate is close to 4.8). In these circumstances, the protonated form of butyrate could diffuse into *Aa*, causing cytoplasmic acidification, and presenting two challenges for *Aa*. First, it would have to overcome excessive butyrate to prevent anion toxicity. Second, it must regulate cytoplasmic protons to prevent prolonged intracellular acidification and maintain intracellular pH homeostasis. The acquisition of the *atoRDAEB* locus and the ability to catabolize butyrate would both help control potential toxicities associated with butyrate, and allow Aa to utilize butyrate as a carbon source, producing acetyl-CoA to be used in the TCA cycle for energy production. This pathway would also produce NADH and FADH_2_ as byproducts, which could fulfil the cell’s bioenergetic needs.

Given the proposed activity of the proteins encoded by the *ato* locus, the activity of the genes encoded by the *hyb* locus may be counterproductive. The Hyb proteins catalyze the oxidation of H_2_ to protons and electrons. The protons are released into the periplasm and electrons are shuttled to quinone’s in the membrane. In the periplasm, the protons themselves may bind and increase butyrate toxicity. Thus, having the Hyb complex is probably of little use when *Aa* can metabolize butyrate, and it may even cause additional toxicities. This situation may have led to a selective pressure to lose the locus, or no selection to maintain it intact.

# References

1. Karched M, Furgang D, Sawalha N, Fine DH. 2012. Rapid identification of oral isolates of Aggregatibacter actinomycetemcomitans obtained from humans and primates by an ultrafast super convection based polymerase chain reaction. J Microbiol Methods 89:71-5.

2. Simpson JT, Wong K, Jackman SD, Schein JE, Jones SJ, Birol I. 2009. ABySS: a parallel assembler for short read sequence data. Genome Res 19:1117-23.

3. Chiu JC, Lee EK, Egan MG, Sarkar IN, Coruzzi GM, DeSalle R. 2006. OrthologID: automation of genome-scale ortholog identification within a parsimony framework. Bioinformatics 22:699-707.

4. Li L, Stoeckert CJ, Jr., Roos DS. 2003. OrthoMCL: identification of ortholog groups for eukaryotic genomes. Genome Res 13:2178-89.

5. Page AJ, Cummins CA, Hunt M, Wong VK, Reuter S, Holden MT, Fookes M, Falush D, Keane JA, Parkhill J. 2015. Roary: rapid large-scale prokaryote pan genome analysis. Bioinformatics 31:3691-3.

6. Brettin T, Davis JJ, Disz T, Edwards RA, Gerdes S, Olsen GJ, Olson R, Overbeek R, Parrello B, Pusch GD, Shukla M, Thomason JA, 3rd, Stevens R, Vonstein V, Wattam AR, Xia F. 2015. RASTtk: a modular and extensible implementation of the RAST algorithm for building custom annotation pipelines and annotating batches of genomes. Sci Rep 5:8365.

7. Overbeek R, Olson R, Pusch GD, Olsen GJ, Davis JJ, Disz T, Edwards RA, Gerdes S, Parrello B, Shukla M, Vonstein V, Wattam AR, Xia F, Stevens R. 2014. The SEED and the Rapid Annotation of microbial genomes using Subsystems Technology (RAST). Nucleic Acids Res 42:D206-14.

8. Aziz RK, Bartels D, Best AA, DeJongh M, Disz T, Edwards RA, Formsma K, Gerdes S, Glass EM, Kubal M, Meyer F, Olsen GJ, Olson R, Osterman AL, Overbeek RA, McNeil LK, Paarmann D, Paczian T, Parrello B, Pusch GD, Reich C, Stevens R, Vassieva O, Vonstein V, Wilke A, Zagnitko O. 2008. The RAST Server: rapid annotations using subsystems technology. BMC Genomics 9:75.

9. Seemann T. 2014. Prokka: rapid prokaryotic genome annotation. Bioinformatics 30:2068-9.

10. Loytynoja A, Goldman N. 2010. webPRANK: a phylogeny-aware multiple sequence aligner with interactive alignment browser. BMC Bioinformatics 11:579.

11. Bouckaert R, Heled J, Kuhnert D, Vaughan T, Wu CH, Xie D, Suchard MA, Rambaut A, Drummond AJ. 2014. BEAST 2: a software platform for Bayesian evolutionary analysis. PLoS Comput Biol 10:e1003537.

12. Jukes TH, Cantor CR. 1969. CHAPTER 24 - Evolution of Protein Molecules, p 21-132. *In* Munro HN (ed), Mammalian Protein Metabolism doi:<https://doi.org/10.1016/B978-1-4832-3211-9.50009-7>. Academic Press.

13. Stamatakis A. 2014. RAxML version 8: a tool for phylogenetic analysis and post-analysis of large phylogenies. Bioinformatics 30:1312-3.

14. Katoh K, Misawa K, Kuma K, Miyata T. 2002. MAFFT: a novel method for rapid multiple sequence alignment based on fast Fourier transform. Nucleic Acids Res 30:3059-66.

15. Fu L, Niu B, Zhu Z, Wu S, Li W. 2012. CD-HIT: accelerated for clustering the next-generation sequencing data. Bioinformatics 28:3150-2.

16. Socransky SS, Dzink JL, Smith CM. 1985. Chemically defined medium for oral microorganisms. J Clin Microbiol 22:303-5.

17. Izano EA, Sadovskaya I, Wang H, Vinogradov E, Ragunath C, Ramasubbu N, Jabbouri S, Perry MB, Kaplan JB. 2008. Poly-N-acetylglucosamine mediates biofilm formation and detergent resistance in Aggregatibacter actinomycetemcomitans. Microb Pathog 44:52-60.

18. Velusamy SK, Sampathkumar V, Godboley D, Fine DH. 2016. Profound Effects of Aggregatibacter actinomycetemcomitans Leukotoxin Mutation on Adherence Properties Are Clarified in in vitro Experiments. PLoS One 11:e0151361.

19. Kachlany SC. 2010. Aggregatibacter actinomycetemcomitans leukotoxin: from threat to therapy. J Dent Res 89:561-70.

20. Yue G, Kaplan JB, Furgang D, Mansfield KG, Fine DH. 2007. A second Aggregatibacter actinomycetemcomitans autotransporter adhesin exhibits specificity for buccal epithelial cells in humans and Old World primates. Infect Immun 75:4440-8.

21. Kachlany SC, Planet PJ, Desalle R, Fine DH, Figurski DH, Kaplan JB. 2001. flp-1, the first representative of a new pilin gene subfamily, is required for non-specific adherence of Actinobacillus actinomycetemcomitans. Mol Microbiol 40:542-54.

22. Kaplan JB, Velliyagounder K, Ragunath C, Rohde H, Mack D, Knobloch JK, Ramasubbu N. 2004. Genes involved in the synthesis and degradation of matrix polysaccharide in Actinobacillus actinomycetemcomitans and Actinobacillus pleuropneumoniae biofilms. J Bacteriol 186:8213-20.

23. Juarez-Rodriguez MD, Torres-Escobar A, Demuth DR. 2013. Construction of new cloning, lacZ reporter and scarless-markerless suicide vectors for genetic studies in Aggregatibacter actinomycetemcomitans. Plasmid 69:211-22.

24. Lee J, Lee HJ, Shin MK, Ryu WS. 2004. Versatile PCR-mediated insertion or deletion mutagenesis. Biotechniques 36:398-400.

25. Didelot X, Walker AS, Peto TE, Crook DW, Wilson DJ. 2016. Within-host evolution of bacterial pathogens. Nat Rev Microbiol 14:150-62.

26. Duchene S, Holt KE, Weill FX, Le Hello S, Hawkey J, Edwards DJ, Fourment M, Holmes EC. 2016. Genome-scale rates of evolutionary change in bacteria. Microb Genom 2:e000094.

27. Wilson AC, Ochman H, Prager EM. 1987. Molecular time scale for evolution. Trends in Genetics 3:241-247.

28. Ochman H, Elwyn S, Moran NA. 1999. Calibrating bacterial evolution. Proceedings of the National Academy of Sciences 96:12638.

29. Ochman H, Wilson AC. 1987. Evolution in bacteria: Evidence for a universal substitution rate in cellular genomes. Journal of Molecular Evolution 26:74-86.

30. Didelot X, Eyre DW, Cule M, Ip CL, Ansari MA, Griffiths D, Vaughan A, O'Connor L, Golubchik T, Batty EM, Piazza P, Wilson DJ, Bowden R, Donnelly PJ, Dingle KE, Wilcox M, Walker AS, Crook DW, Peto TE, Harding RM. 2012. Microevolutionary analysis of Clostridium difficile genomes to investigate transmission. Genome Biol 13:R118.

31. Wilson DJ, Gabriel E, Leatherbarrow AJ, Cheesbrough J, Gee S, Bolton E, Fox A, Hart CA, Diggle PJ, Fearnhead P. 2009. Rapid evolution and the importance of recombination to the gastroenteric pathogen Campylobacter jejuni. Mol Biol Evol 26:385-97.

32. Morelli G, Didelot X, Kusecek B, Schwarz S, Bahlawane C, Falush D, Suerbaum S, Achtman M. 2010. Microevolution of Helicobacter pylori during Prolonged Infection of Single Hosts and within Families. PLOS Genetics 6:e1001036.

33. Biek R, Pybus OG, Lloyd-Smith JO, Didelot X. 2015. Measurably evolving pathogens in the genomic era. Trends Ecol Evol 30:306-13.

34. Haubek D, Poulsen K, Kilian M. 2007. Microevolution and patterns of dissemination of the JP2 clone of Aggregatibacter (Actinobacillus) actinomycetemcomitans. Infect Immun 75:3080-8.

35. Ramsey MM, Rumbaugh KP, Whiteley M. 2011. Metabolite cross-feeding enhances virulence in a model polymicrobial infection. PLoS Pathog 7:e1002012.

36. Stacy A, Everett J, Jorth P, Trivedi U, Rumbaugh KP, Whiteley M. 2014. Bacterial fight-and-flight responses enhance virulence in a polymicrobial infection. Proceedings of the National Academy of Sciences 111:7819.

37. Fine DH, Patil AG, Velusamy SK. 2019. Aggregatibacter actinomycetemcomitans (Aa) Under the Radar: Myths and Misunderstandings of Aa and Its Role in Aggressive Periodontitis. Frontiers in Immunology 10.
