# Supplementary material for: Adaptation by Ancient Horizontal Acquisition of Butyrate Metabolism Genes in Aggregatibacter actinomycetemcomitans
Source: mBio. 2021 Mar 23;12(2):e03581-20. doi: 10.1128/mBio.03581-20 (PMC8092312; doi:10.1128/mBio.03581-20)
Supplement: FIG S1 [file mBio.03581-20-sf001.docx]

Pangenome analysis of 95 *Aa* isolates from primates. **A.** A pie chart of the pan genome, breaking down the genes into core, soft core, shell and cloud and showing the number of isolates they are present in. **B.** A table showing the genes that are unique to the two major clades (Human and Monkey) in the tree of Figure 1A. **C.** Illustration of the genes of the two loci showing the size of each gene in base pair (bp) and the size of proteins in KDa.
