# Supplementary material for: Adaptation by Ancient Horizontal Acquisition of Butyrate Metabolism Genes in Aggregatibacter actinomycetemcomitans
Source: mBio. 2021 Mar 23;12(2):e03581-20. doi: 10.1128/mBio.03581-20 (PMC8092312; doi:10.1128/mBio.03581-20)
Supplement: FIG S2 [file mBio.03581-20-sf002.docx]

Phylogenetic analysis of the *H. influenzae* species showing the presence/absence of the *atoRDAEB* locus and capsule*.* Maximum likelihood phylogenetic tree constructed with 755 genomes from GenBank. First Ring and Second Ring from inside to outside represent *atoRDAEB* locus and capsule, respectively. All capsulated genomes has the *atoRDAEB* locus. The tree was edited using iTOL website (v4.2.3). The tree scale indicates the number of substitutions per site.
