# Supplementary material for: Adaptation by Ancient Horizontal Acquisition of Butyrate Metabolism Genes in Aggregatibacter actinomycetemcomitans
Source: mBio. 2021 Mar 23;12(2):e03581-20. doi: 10.1128/mBio.03581-20 (PMC8092312; doi:10.1128/mBio.03581-20)
Supplement: FIG S3 [file mBio.03581-20-sf003.docx]

Phylogenetic analysis of the *atoDAEB* locus in the *Pasteurellaceae* family with outgroup species from the *Gammaproteobacteria*. Maximum likelihood tree of the *atoDAEB* locus in the *Pasteurellaceae* family using RAxML with Gammaproteobacteria species *Y. intermedia*, *M. morganii* and *Erwinia teleogrylli* were included as outgroups. The root was determined on the branch that makes *Pasteurellaceae family as ingroup* and other members from the *Gammaproteobacteria* as an outgroup. Bootstrap values are reported on all branches. Species clades with more than four clusters were collapsed for easier visualization. The alignment was limited to *atoDAEB, atoR* was excluded as it is absent outside of *Pasteurellaceae* family*.* The sequences were clustered using CD-HIT (100% identity and coverage). All clusters were composed of individual species. The total number of genomes in each cluster and sequence length are shown respectively and separated by underscore in the nodes that were not collapsed.
