# Supplementary material for: Adaptation by Ancient Horizontal Acquisition of Butyrate Metabolism Genes in Aggregatibacter actinomycetemcomitans
Source: mBio. 2021 Mar 23;12(2):e03581-20. doi: 10.1128/mBio.03581-20 (PMC8092312; doi:10.1128/mBio.03581-20)
Supplement: TABLE S2 [file mBio.03581-20-st002.docx]

**Media’s pH after adding dextrose and butyrate as carbon sources.**

|  |  | Initial pH | pH after 48 hours |
| --- | --- | --- | --- |
| IDH781 | Dextrose | 7.10 | 5.61 |
|  | Butyrate | 7.17 | 6.80 |
