# Supplementary material for: Adaptation by Ancient Horizontal Acquisition of Butyrate Metabolism Genes in Aggregatibacter actinomycetemcomitans
Source: mBio. 2021 Mar 23;12(2):e03581-20. doi: 10.1128/mBio.03581-20 (PMC8092312; doi:10.1128/mBio.03581-20)
Supplement: TABLE S3 [file mBio.03581-20-st003.docx]

**Primers used in the deletion of the *ato* locus.**

| Primer | Purpose | Sequence |
| --- | --- | --- |
| atoNotIUF | Fusion PCR | GGGCCCAATTAATGGCGACCAATAGAACCAAGGACACGATGTG |
| atoUR | Fusion PCR | CATTGTTTTATTCGACGGAATCCTTGGAGTGTAACCTTG |
| atoDF | Fusion PCR | CAAGGTTACACTCCAAGGATTCCGTCGAATAAAACAATG |
| atoXhoIDR | Fusion PCR | TACTAGTTCGAATAACAGACATTGTGGAATCCTTTACCGAAGG |
| atoOUTF | Confirmation of *ato* mutant | CGCTCAGCATCTACACGAACAACTTCAAGTG |
| atoOUTR | Confirmation of *ato* mutant | CTTATCCTGATTTTATTCTGTATCGCGTTCC |
| pSV7SEQF1 | Internal primers for sequencing | CATATTGGTCACGCGCATCTTTG |
| pSV7SEQF2 | Internal primers for sequencing | CGCTGCAATGCCTTAAGAATGCG |
